# WHAT IS IN A NAME: DEMENTIA AND CAREGIVERS IN THE AFRICAN IMMIGRANT COMMUNITY

**DOI:** 10.1093/geroni/igae098.1149

**Published:** 2024-12-31

**Authors:** Manka Nkimbeng, Truphosa Aswani, Joseph Gaugler

**Affiliations:** University of Minnesota, Minneapolis, Minnesota, United States; University of Minnesota, Minneapolis, Minnesota, United States; University of Minnesota, Minneapolis, Minnesota, United States

## Abstract

African immigrants are among the racial/ethnic minority groups whose experiences of dementia are not fully understood. However, the use of appropriate terminology is necessary to facilitate appropriate outreach and research with the African immigrant community. The purpose of this qualitative study is to describe how African immigrants define dementia and care partners. Thematic analysis of transcripts from ten key informant interviews (with leaders including priests, executive directors, etc.) and three community conversations (30 participants). Themes describe that there are no accepted words for dementia or caregiver in many African immigrant languages. Translations for dementia are often based on the symptoms, other derogatory terminology, and myths. The concept of a caregiver or care partner is also unfamiliar to the community, even when individuals are performing such roles. The language surrounding dementia care significantly impacts healthcare access and quality care, especially for multilingual and multicultural communities. Identifying and clarifying these terminologies are a key component of outreach and education that is necessary to decrease the risk and burden of dementia in these communities that are disproportionately impacted.

